# Comparative Microbiome Analysis of Three Epidemiologically Important Tick Species in Latvia

**DOI:** 10.3390/microorganisms11081970

**Published:** 2023-07-31

**Authors:** Agne Namina, Alisa Kazarina, Marija Lazovska, Sarmite Akopjana, Viktorija Ulanova, Agnija Kivrane, Lauma Freimane, Darja Sadovska, Janis Kimsis, Antra Bormane, Valentina Capligina, Renate Ranka

**Affiliations:** Latvian Biomedical Research and Study Centre, Ratsupites Street 1, k-1, LV-1067 Riga, Latvia; agne.namina@gmail.com (A.N.); alisa.kazarina@biomed.lu.lv (A.K.); marija.lazovska@inbox.lv (M.L.); sarmite.kup@inbox.lv (S.A.); viktorija.igumnova@biomed.lu.lv (V.U.); agnija.kivrane@biomed.lu.lv (A.K.); lauma.veidemane@biomed.lu.lv (L.F.); darja.aleinikova@biomed.lu.lv (D.S.); janiskimsis@gmail.com (J.K.); antra.bormane@gmail.com (A.B.); capligina@yahoo.com (V.C.)

**Keywords:** tick microbiome, *Ixodes ricinus*, *Ixodes persulcatus*, *Dermacentor reticulatus*, Latvia

## Abstract

(1) Background: Amplicon-based 16S rRNA profiling is widely used to study whole communities of prokaryotes in many niches. Here, we comparatively examined the microbial composition of three tick species, *Ixodes ricinus*, *Ixodes persulcatus* and *Dermacentor reticulatus*, which were field-collected in Latvia. (2) Methods: Tick DNA samples were used for microbiome analysis targeting bacterial 16S rDNA using next-generation sequencing (NGS). (3) Results: The results showed significant differences in microbial species diversity and composition by tick species and life stage. A close similarity between microbiomes of *I. ricinus* and *I. persulcatus* ticks was observed, while the *D. reticulatus* microbiome composition appeared to be more distinct. Significant differences in alpha and beta microbial diversity were observed between *Ixodes* tick life stages and sexes, with lower taxa richness indexes obtained for female ticks. The *Francisella* genus was closely associated with *D. reticulatus* ticks, while endosymbionts *Candidatus Midichlorii* and *Candidatus Lariskella* were associated with *I. ricinus* and *I. persulcatus* females, respectively. In *I. ricinus* females, the endosymbiont load negatively correlated with the presence of the *Rickettsia* genus. (4) Conclusions: The results of this study revealed important associations between ticks and their microbial community and highlighted the microbiome features of three tick species in Latvia.

## 1. Introduction

Ticks are important vectors of pathogens that affect both humans and animals worldwide. In addition to the pathogens they transmit, ticks harbor a diverse group of commensals and symbiotic microorganisms that collectively comprise the tick microbiome [1]. The development of high-throughput sequencing technologies has enabled the in-depth microbiome profiling of metagenomic samples, and is widely used nowadays to study whole communities of prokaryotes in many niches [2]. During the last decade, the microbiome of several tick species belonging to the genera *Ixodes*, *Dermacentor*, *Amblyomma, Hyalomma*, *Haemaphysalis* and *Rhipicephalus* has been analyzed, and the obtained information has altered our understanding of tick–microbe interactions [3]. In particular, microbiome studies have highlighted the complexity and dynamic variability of tick microbial communities, as well as the significance of the tick microbiota in tick biology [1,3]. It was shown that tick symbionts and commensals can play various roles in nutritional adaptation, development, reproduction, defense against environmental stress and immunity, as well as in vector competence and pathogen transmission dynamics [4,5,6].

Recent studies have increasingly shown that the tick microbiome varies by geographical origin, species, sex, life stages, environmental stress, tick immunity, host and blood meal [7,8,9,10,11,12,13,14]. In particular, the results showed that tick-associated bacterial communities are largely species-specific, and microbiota of nymphs and males appeared to be more diverse than those of adult females [7,9,10,12]. The findings of several studies revealed that tick samples originating from geographically close locations had shown higher microbiome similarity [11,12,13,14]. In addition, the characterization of the bacterial microbiota in *Ixodes ricinus* ticks along a replicated elevational gradient revealed a lower variation in microbial community composition at higher elevations; also, a higher microbial diversity later in the season was reported [9].

Studying the tick microbiome is also a critical step in understanding how tick-borne diseases are transmitted, and how to prevent them. By studying and understanding the tick microbiome, we can identify environmental factors that promote the growth of disease-causing bacteria, which, in turn, may potentially lead to the development of new treatments and preventatives. To address this question, the concepts of scale and temporality were highlighted as crucial when studying tick microbial communities, as it allows for achieving better understanding of the tick microbial community ecology and pathogen/microbiota interactions [3]. In Latvia, a Baltic state in northern Europe, three epidemiologically important tick species are present: *I. ricinus* is widespread throughout the territory, while *Ixodes persulcatus* is restricted to the Latgale and eastern and northeastern Vidzeme regions, and *Dermacentor reticulatus* is located in the western, southern and central regions of Latvia, including the Riga region [15]. Despite *Ixodes* and *Dermacentor* ticks being of great public health relevance, their microbial communities, apart from intensively studied tick-borne infections, are still largely unexplored. In this study, we aimed to compare the bacterial communities present within Latvian *I. ricinus*, *I. persulcatus* and *D. reticulatus* ticks under natural field conditions by using 16S rRNA high-throughput sequencing technology. Both immature and adult ticks of all three species were studied, and microbiome data were explored in the context of ecological studies.

## 2. Materials and Methods

### 2.1. Sampling Design and Tick Sampling 

Ticks were collected using the flagging method in different regions according to the tick species distribution and sympatric area patterns in Latvia [15] in order to avoid location-dependent biases in microbiome analysis. The collection sites were geolocated and maps were created using the Google Earth platform (http://www.google.co.uk/intl/en_uk/earth (accessed on 27 July 2022) (Supplementary Figure S1)). Tick samples were preserved in 70% ethanol. Ticks were identified to the species level, and their stage and sex were identified based on morphological characteristics [16,17] After morphological identification, the ticks were individually stored at −20 °C.

### 2.2. DNA Extraction and Tick Species Identification

DNA was extracted via the phenol/chloroform method as described previously [15]. In total, 126 field-collected tick samples were studied: 53 *I. ricinus*, 40 *I. persulcatus* and 33 *D. reticulatus* ticks. Tick species of the samples were confirmed using the quantitative real-time PCR (qPCR) method based on the detection of the ITS2 gene. Primers and probes were described previously [18]. For *I. ricinus* and *I. persulcatus* species identification, a multiplex reaction was performed in a final volume of 12 μL containing TaqMan™ Fast Advanced Master Mix (Applied Biosystems, Waltham, MA, USA), 150 nM of each primer, 50 nM of *I. ricinus* probe, 100 nM of *I. persulcatus* probe and 2 μL of tick DNA. Thermal cycling conditions were as follows: 50 °C for 2 min, 95 °C for 20 s, followed by 40 cycles of a 2-step amplification profile at 95 °C for 3 s and 65 °C for 30 s. For the *D. reticulatus* species identification, qPCR mix contained TaqMan™ Fast Advanced Master Mix (Applied Biosystems, USA), 280 nM of each primer and probe and 2 μL of control DNA. Thermal cycling conditions were as follows: 50 °C for 2 min, 95 °C fo 3 min, followed by 40 cycles of a 2-step amplification profile of 10 s at 95 °C and 20 s at 60 °C. Negative controls (nuclease-free water instead of DNA) were included in every reaction. The reactions were performed using a ViiA 7 real-time PCR system (Thermo Fisher Scientific, Waltham, MA, USA). The results were analyzed using QuantStudio software (Thermo Fisher Scientific, USA).

### 2.3. Library Preparation and Sequencing

Extracted DNA underwent the process of amplicon library preparation using Ion 16STM Metagenomics Kit (Life Technologies, Carlsbad, CA, USA) following manufacturer instructions. Two sets of primers for the amplification of bacterial 16S rRNA were used: the first primer set was used to amplify 16S rRNA variable regions 2–4–8 (V 2–4–8), whereas the second primer set targeted 16S rRNA variable regions 3–6 and 7–9 (V 3–6; V 7–9). Prior to sequencing, the obtained amplicons of both primer sets were combined together for each sample. Immediately after preparation, libraries were examined for size, quality and concentration using Agilent High-Sensitivity DNA Kit and Bioanalyser 2100 instrument (Agilent Technologies, Santa Clara, CA, USA).

16S rRNA amplicon sequencing was performed using the Ion PGM System and 318 v2 chip (Thermo Fisher Scientific, Waltham, MA, USA) according to the manufacturer instructions.

To control laboratory contamination, tick 16S rRNA amplicon sequencing procedures were accompanied by corresponding blank samples, which were also treated equally and underwent the same library preparation steps. However, blank control samples showed no detectable amplification (Supplementary Figure S2).

Raw sequencing reads have been submitted to the European Nucleotide Archive, project accession PRJEB63277.

### 2.4. 16S rRNA Amplicon Sequence Analysis

Sequencing data were further analyzed with a variety of computational methods including freely available programs and in-house scripts. Sequencing data preprocessing on the local Ion Torrent Proton server included initial quality control steps as well as data assignment to each individual sample. Barcodes and sequencing adapters, together with polyclonal and low-quality sequences, were filtered by Proton software during the first post-sequencing data handling step. The resultant data were exported for further manipulations in the form of BAM files. First-stage quality control was performed using Galaxy Public Server, overrepresented adapter sequences were removed, and sequencing reads were filtered based on quality score, i.e., sequences with PHRED quality score <20 were excluded from further manipulations [19,20]. Taxonomic profiling was performed using Parallel-META 3 using Greengenes 13_8 (16S rRNA, 97% level) database [21].

### 2.5. Statistical Analysis

Visual representation, abundance, diversity and statistical analysis of tick microbiome samples were performed using Microbiome Analyst online server (https://www.microbiomeanalyst.ca/) [22,23]. The analysis was run for all samples together, as well as for each tick species separately. For alpha diversity analysis, Chao 1 index was used to determine differences between the sample groups. Beta diversity of the samples was assessed by principal coordinate analysis (PCoA) and permutational multivariate analysis of variance (PERMANOVA) to show microbial composition of the sample groups. Dendrogram analysis using the Bray–Curtis Index and the Ward clustering method was used to determine the separation of the samples. Additionally, the Linear Discriminant Analysis Effect Size (LefSe) analysis was performed.

## 3. Results

### 3.1. Tick Sample Characteristics

In total, 126 tick samples were included in this study: 53 samples were *I. ricinus*, 40 were *I. persulcatus* and 33 were *D. reticulatus* (Table 1). For each tick species, samples of unfed male, female and nymph ticks were analyzed. Tick samples were collected in two biotopes: in a woodland/grassy area ecotone (N = 70) and in a mixed forest (N = 56). The vast majority of *D. reticulatus* ticks were found in woodland/grassy area ecotones (87.9%) (Table 1).

### 3.2. Microbial Diversity and Microbiome Composition of Three Tick Species

**Figure 1:**
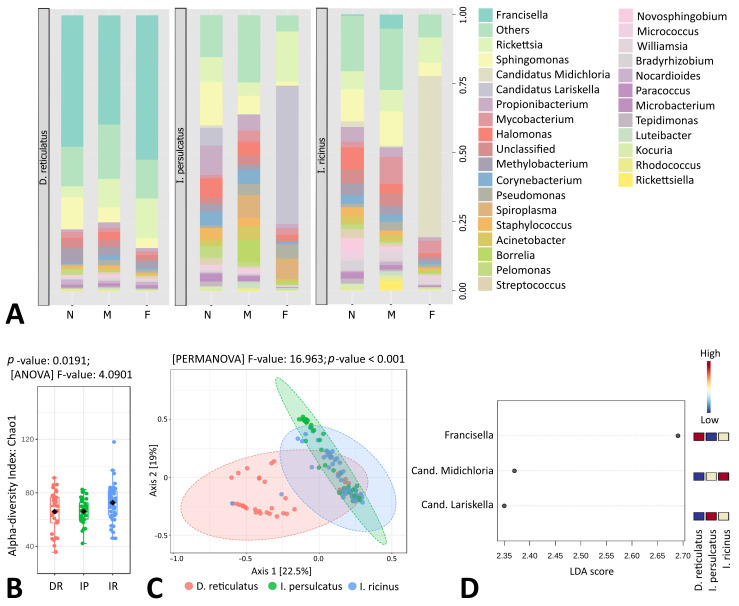
Genus-level comparison of bacterial profiles in *Ixodes ricinus*, *I. persulcatus* and *Dermacentor reticulatus* tick samples: (**A**) Stacked plots of the taxonomic classification. The abundances of the most abundant genera are shown. N: nymph, F: female, M: male. (**B**) Chao1 diversity analysis. DR: *D. reticulatus*, IP: *I. persulcatus*, IR: *I. ricinus*. (**C**) Principal coordinate analysis (PCoA) derived from Bray–Curtis distances among samples of the three groups (*p* < 0.001 by PERMANOVA). For each axis, in square brackets, the percent of variation explained was reported. (**D**) Linear discriminant analysis (LDA) combined with effect size measurements (LEfSe) identified microbial genera that enabled discrimination between the microbiotas of three tick species. False Discovery Rate (FDR)-adjusted *p*-value cutoff: 0.05; logarithmic LDA score ≥ 2.0.

For tick samples, on average, 85769 taxonomically assigned reads were obtained per sample at the genus level; the maximum and minimum read count were 954453 and 2083 reads, respectively (Supplementary Figure S3). In total, at the genus level, 96 OTUs were discovered within the samples. For further analysis, all samples were rarefied to even sequencing depth. Among all tick samples, ten of the most abundant microbial genera were Ca. *Lariskella*, Ca. *Midichloria*, *Corynebacterium*, *Francisella*, *Halomonas*, *Methylobacterium*, *Mycobacterium*, *Propionibacterium*, *Pseudomonas*, *Rickettsia* and *Sphingomonas*; however, significant differences in microbial species diversity and composition by tick species, sex and life stage were observed (Figure 1A).

Alpha diversity analysis by Chao1 index showed a statistically significant difference between three sample groups based on tick species (*p* = 0.0191), and *I. ricinus* tick samples appeared to have the greatest taxonomical diversity (Figure 1B). The results of beta diversity analysis by principal coordinate analysis (PCoA) and permutational multivariate analysis of variance (PERMANOVA) showed that the microbial composition of three tick species was significantly different (*p* < 0.001), with *I. ricin*us and *I. persulcatus* sample clusters being more related to each other, while *D. reticulatus* samples formed a more distant group (Figure 1C). Also, a dendrogram analysis using the Bray–Curtis Index and the Ward clustering method showed, with a few exceptions, a separation of the samples based of the tick species, sex and developmental stage (Supplementary Figure S4).

Based on the linear discriminant analysis effect size (LefSe) analysis, three microbial taxa with significantly different (*p* < 0.05) abundance were detected among the three tick species (Figure 1D). Among these genera, Ca. *Lariskella* was more abundant in *I. persulcatus*, Ca. *Midichloria*—in *I. ricinus*, and *Francisella*—in *D. reticulatus* ticks.

### 3.3. Microbiome Composition of I. ricinus Ticks

**Figure 2:**
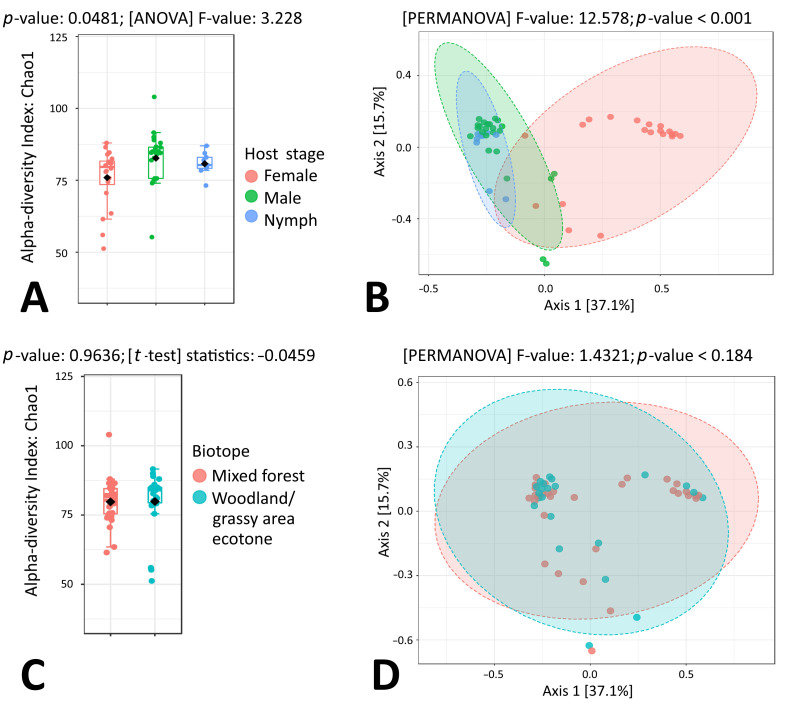
Genus-level comparison of bacterial profiles in *I. ricinus* tick samples: (**A**) Chao1 diversity analysis of nymph, male and female samples. (**B**) PCoA derived from Bray–Curtis distances among tick samples of the three groups: nymphs, males and females (*p* < 0.001 by PERMANOVA). (**C**) Chao1 diversity analysis of tick samples from different biotopes. (**D**) PCoA derived from Bray–Curtis distances among tick samples of the two biotope groups (*p*-value by PERMANOVA).

When taking a closer look at microbial taxonomic data in the context of individual tick species, a statistically significant difference between *I. ricinus* sex groups was observed for both alpha and beta microbial diversity (*p* = 0.0481 and *p* < 0.001, respectively) (Figure 2A,B). On the other hand, no differences were observed for microbiome composition when tick samples representing mixed forest and woodland/grassy area ecotones were compared (Figure 2C,D). In *I. ricinus* nymphs, ten of the most abundant microbial genera were *Sphingomonas* (11.67%), *Halomonas* (7.80%), *Rickettsia* (6.52%), *Propionibacterium* (5.30%), *Novosphingobium* (4.92%), *Methylobacterium* (4.21%), *Bradyrhizobium* (3.56%), *Staphylococcus* (3.36%), *Corynebacterium* (3.16%) and *Streptococcus* (3.16%) (Supplementary Table S1). Quite similarly, in *I. ricinus* males, ten of the most abundant microbial genera were *Sphingomonas* (12.44%), *Mycobacterium* (9.85%), *Rickettsia* (7.82%), *Francisella* (5.14%), *Rickettsiella* (3.45%), *Propionibacterium* (3.44%), *Halomonas* (3.37%), *Methylobacterium* (3.26%), *Pseudomonas* (3.14%) and *Williamsia* (2.86%) (Supplementary Table S1). The results of beta diversity analysis by PCoA and PERMANOVA showed that *I. ricinus* nymph and male samples formed tightly overlapping clusters, while *I. ricinus* female samples formed a distant group (Figure 2B). Indeed, in *I. ricinus* females, the most abundant microbial genus Ca. *Midichloria* comprised more than half of all sequencing reads (58.38%), followed by *Rickettsia* (9.12%), *Sphingomonas* (4.88%), *Mycobacterium* (4.59%), *Williamsia* (2.31%), *Propionibacterium* (1.25%), *Methylobacterium* (1.20%), *Pseudomonas* (1.00%), *Acinetobacter* (0.96%) and *Corynebacterium* (0.92%) (Figure 1A, Supplementary Table S1).

**Figure 3:**
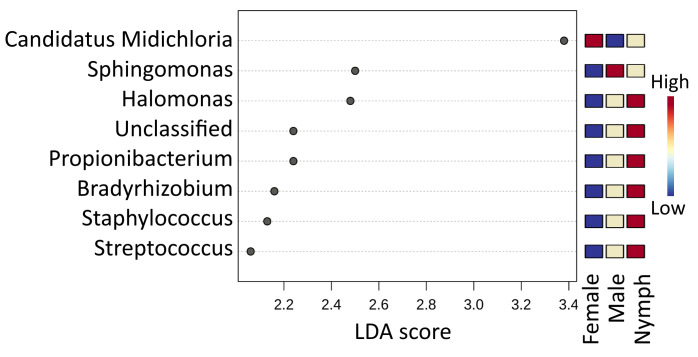
LEfSe identified microbial genera that enabled discrimination between the microbiotas of *I. ricinus* nymph, male and female samples. False Discovery Rate (FDR)-adjusted *p*-value cutoff: 0.05; logarithmic LDA score ≥ 2.0.

LefSe analysis revealed seven microbial taxa with significantly different (*p* < 0.05) abundance: Ca. *Midichloria* was more abundant in females and *Sphingomonas* was more abundant in males, while *Halomonas*, *Propionibacterium*, *Bradyrhizobium*, *Staphylococcus* and *Streptococcus* were highly associated with *I. ricinus* nymphs (Figure 3).

### 3.4. Microbiome Composition of I. persulcatus Ticks

**Figure 4:**
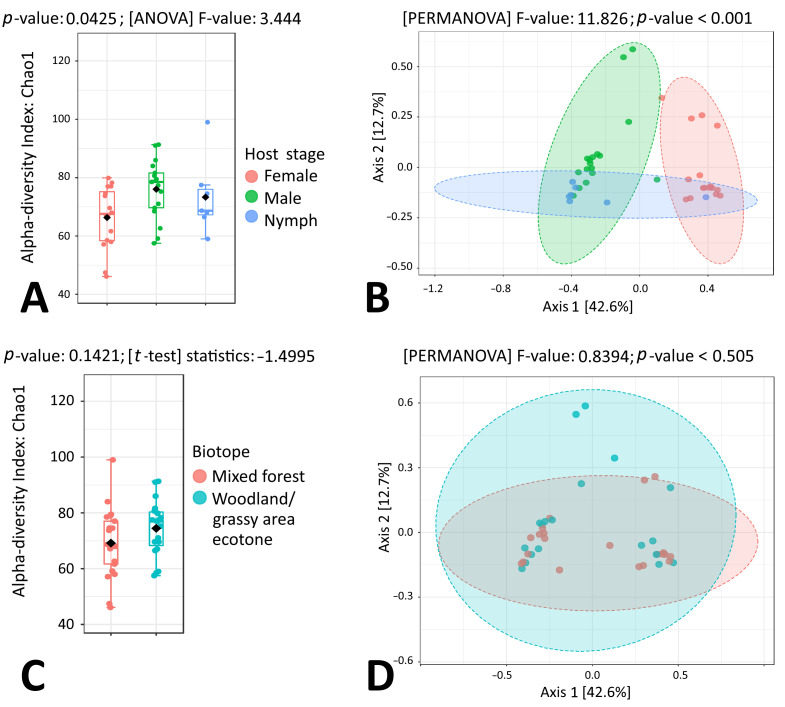
Genus-level comparison of bacterial profiles in *I. persucatus* tick samples: (**A**) Chao1 diversity analysis of nymph, male and female samples. (**B**) PCoA derived from Bray–Curtis distances among tick samples of the three groups: nymphs, males and females (*p* < 0.001 by PERMANOVA). (**C**) Chao1 diversity analysis of tick samples from different biotopes. (**D**) PCoA derived from Bray–Curtis distances among tick samples of the two biotope groups (*p*-value by PERMANOVA).

Similarly to *I. ricinus*, a statistically significant difference between *I. persulcatus* sex and development stage groups was observed for both alpha and beta microbial diversity (*p* = 0.0425 and *p* < 0.001, respectively), while no differences were detected for microbiome composition when tick samples representing mixed forest and woodland/grassy area ecotones were compared (Figure 4A–D). The results of beta diversity analysis by PCoA and PERMANOVA showed that *I. persulcatus* nymph, male and female samples formed three clusters (Figure 4B).

**Figure 5:**
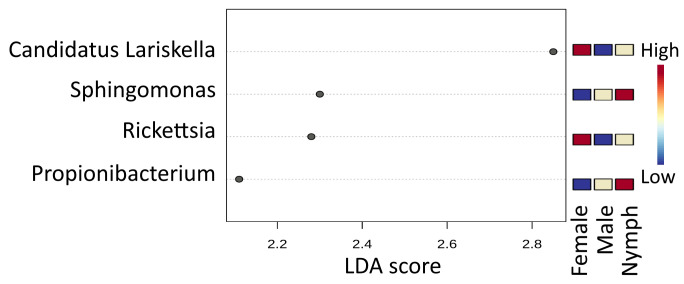
LEfSe identified microbial genera that enabled discrimination between the microbiotas of *I. persulcatus* nymph, male and female samples. False Discovery Rate (FDR)-adjusted *p*-value cutoff: 0.05; logarithmic LDA score ≥ 2.0.

In *I. persulcatus* nymphs, ten of the most abundant microbial genera were *Sphingomonas* (15.81%), *Propionibacterium* (10.62%), *Rickettsia* (8.90%), *Halomonas* (7.00%), Ca. *Lariskella* (6.43%), *Corynebacterium* (4.90%), *Pelomonas* (4.31%), *Staphylococcus* (4.06%), *Methylobacterium* (2.93%) and *Streptococcus* (2.48%). In *I. persulcatus* males, ten of the most abundant microbial genera were *Spiroplasma* (8.32%), *Borrelia* (7.88%), *Sphingomonas* (6.65%), *Propionibacterium* (5.71%), *Acinetobacter* (5.15%), *Corynebacterium* (5.15%), *Halomonas* (5.00%), *Rickettsia* (4.84%), *Pseudomonas* (4.04%) and *Mycobacterium* (4.01%). In *I. persulcatus* females, ten of the most abundant microbial genera were Ca. *Lariskella* (50.07%), *Rickettsia* (18.19%), *Spiroplasma* (7.41%), *Pseudomonas* (5.18%), *Mycobacterium* (2.40%), *Propionibacterium* (1.58%), *Borrelia* (1.40%), *Sphingomonas* (1.40%), *Halomonas* (1.21%) and *Luteibacter* (0.81%). LefSe analysis revealed four microbial taxa with significantly different (*p* < 0.05) abundance: Ca. *Lariskella* and *Rickettsia* were more abundant in females, and *Sphingomonas* and *Propionibacterium* were more abundant in *I. persulcatus* nymphs (Figure 5).

### 3.5. Microbiome Composition of D. reticulatus Ticks

**Figure 6:**
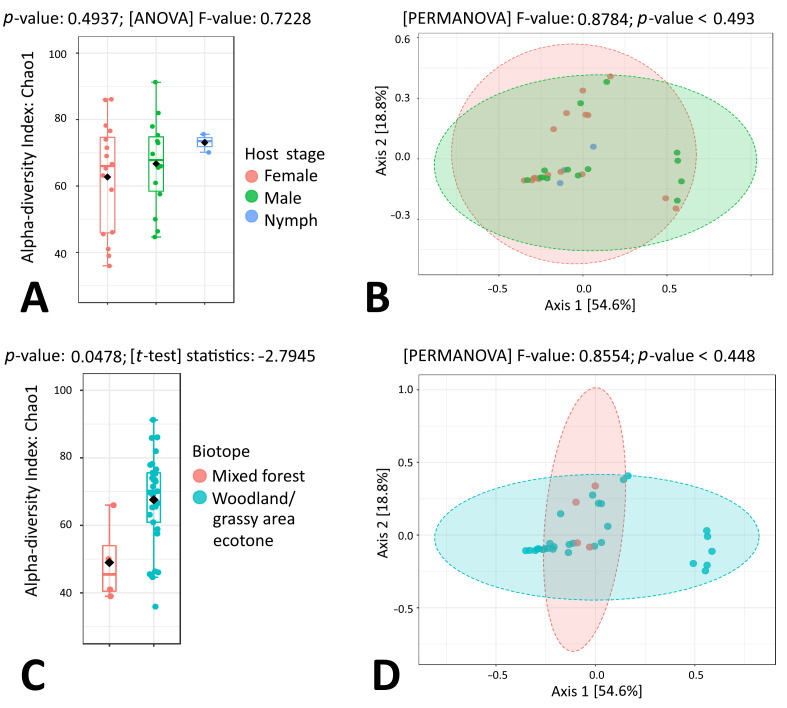
Genus-level comparison of bacterial profiles in *D. reticulatus* tick samples: (**A**) Chao1 diversity analysis of nymph, male and female samples. (**B**) PCoA derived from Bray–Curtis distances among tick samples of the three groups: nymphs, males and females (*p*-value by PERMANOVA). (**C**) Chao1 diversity analysis of tick samples from different biotopes. (**D**) PCoA derived from Bray–Curtis distances among tick samples of the two biotope groups (*p*-value by PERMANOVA).

In contrast to the *Ixodes* species, in this study, a statistically significant difference between *D. reticulatus* sex and development stage groups was not observed for alpha and beta microbial diversity (*p* > 0.05) (Figure 6A,B). However, the microbial alpha diversity of the *D. reticulatus* ticks collected in a woodland/grassy area ecotone was slightly higher than for the samples representing mixed forest areas, and this difference reached statistical significance (*p* = 0.0478) (Figure 6C). The results of beta diversity analysis by PCoA and PERMANOVA showed that *D. reticulatus* samples from both biotopes formed overlapped clusters (*p* = 0.448; Figure 6D).

In *D. reticulatus* nymphs, ten of the most abundant microbial genera were *Francisella* (47.66%), *Sphingomonas* (11.67%), *Methylobacterium* (5.12%), *Rickettsia* (3.94%), *Mycobacterium* (2.16%), *Pelomonas* (1.95%), *Nocardioides* (1.81%), *Propionibacterium* (1.01%), *Jatrophihabitans* (0.96%) and *Sphingomonadaceae* Group (0.85%). In *D. reticulatus* males, ten of the most abundant microbial genera were *Francisella* (39.57%), *Rickettsia* (10.29%), *Sphingomonas* (5.42%), *Methylobacterium* (3.18%), *Halomonas* (2.63%), *Propionibacterium* (1.98%), *Pseudomonas* (1.90%), *Nesterenkonia* (1.84%), *Corynebacterium* (1.71%) and *Mycobacterium* (1.58%). Quite similarly, in *D. reticulatus* females, ten of the most abundant microbial genera were *Francisella* (52.30%), *Rickettsia* (14.30%), *Sphingomonas* (3.64%), *Methylobacterium* (2.51%), *Mycobacterium* (1.30%), Ca. *Endoecteinascidia* (1.23%), *Haemophilus* (1.22%), *Propionibacterium* (1.20%), *Nocardioides* (1.20%) and *Pseudomonas* (0.99%) (Figure 1A, Supplementary Table S1). Accordingly, no differentially abundant microbial taxa were identified between *D. reticulatus* tick sample groups.

### 3.6. Endosymbiont Species Abundance in Female Ixodes Ticks

**Figure 7:**
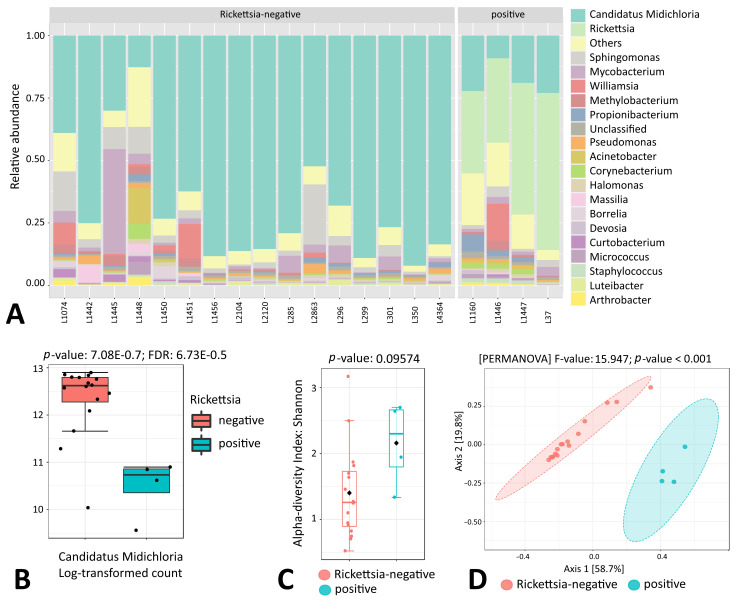
Genus-level comparison of bacterial profiles in *I. ricinus* female tick samples: (**A**) Stacked plots of the taxonomic classification. The abundances of the most abundant genera are shown. (**B**) Comparison of the relative abundance of Ca. *Midichloria* between *Rickettsia*-positive and -negative samples. *p* value is indicated. (**C**) Shannon diversity analysis of *Rickettsia*-positive and -negative samples. (**D**) PCoA derived from Bray–Curtis distances among *Rickettsia*-positive and -negative samples (*p* < 0.001 by PERMANOVA).

In *I. ricinus*, endosymbiont Ca. *Midichloria* was detected almost exclusively in female tick samples; only a small portion of sequencing reads of male ticks and nymphs were attributed to this genus (0.58% and 2.04%, respectively) (Supplementary Table S1). The prevalence of Ca. *Midichloria* in *I. ricinus* female ticks was 100%; however, the number of endosymbiont reads was not uniform and, as appeared, was largely dependent on the presence of the *Rickettsia* genus (Figure 7A,B). Further analysis indicated statistically significant differences in microbial beta diversity between *Rickettsia*-positive and -negative *I. ricinus* females (*p* < 0.001), while the alpha diversity was not affected (Figure 7C,D). 

**Figure 8:**
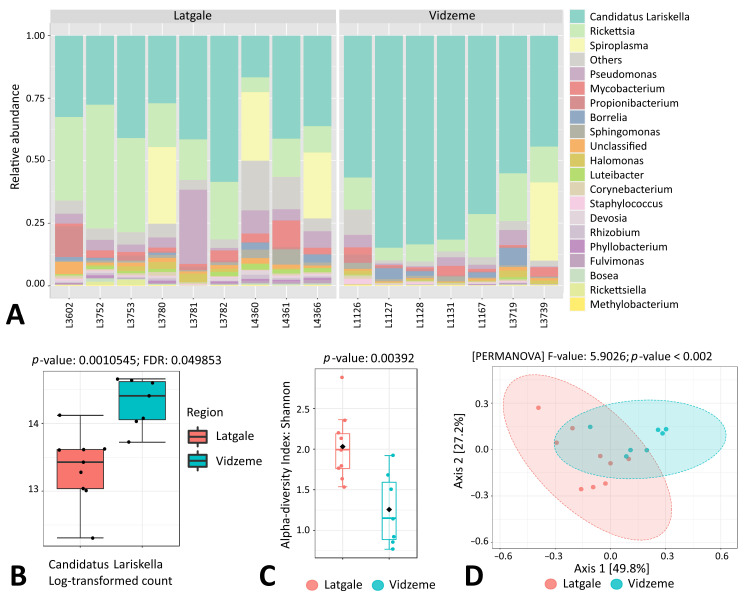
Genus-level comparison of bacterial profiles in *I. persulcatus* female tick samples: (**A**) Stacked plots of the taxonomic classification. The abundances of the most abundant genera are shown. (**B**) Comparison of the relative abundance of Ca. *Lariskella* between samples from Latgale and Vidzeme regions. *p* value is indicated. (**C**) Shannon diversity analysis of samples from Latgale and Vidzeme regions. (**D**) PCoA derived from Bray–Curtis distances among samples from Latgale and Vidzeme regions (*p* < 0.001 by PERMANOVA).

Similarly, in *I. persulcatus*, endosymbiont Ca. *Lariskella* was mainly associated with female ticks, as only a small fraction of sequencing reads in male and nymph ticks belonged to this genus (50.07% versus 0.13% and 6.43%, respectively) (Supplementary Table S1). However, in contrast to *I. ricinus*, all *I. persulcatus* females appeared to be *Rickettsia*-positive in this study; thus, the presence of *Rickettsia* alone did not explain the variability in the endosymbiont load observed in the *I. persulcatus* female samples (Figure 8A). On the other hand, the Ca. *Lariskella* genus was significantly more abundant in *I. persulcatus* females collected in the Vidzeme region in comparison to the Latgale region (Figure 8A,B). In addition, statistically significant differences in both alpha and beta microbial diversity were observed between Vidzeme and Latgale *I. persulcatus* female ticks (Figure 8C,D). On the contrary, for *I. ricinus* females, we did not observe any relations between tick collection site (i.e., geographical region or biotope) and the number of endosymbionts in this study.

## 4. Discussion

In Latvia, *I. ricinus* is widespread throughout the territory, while *I. persulcatus* is present in the eastern region and *D. reticulatus* is located in the western and southern regions; thus, sympatric populations of *D. reticulatus* and *I. ricinus* ticks, as well as *D. reticulatus*, *I. ricinus* and *I. persulcatus* ticks, exist in the country [15]. Despite the sympatric occurrence of the tick species, the obtained results clearly indicated the diversity in tick microbiome composition: while a close similarity between the microbiome of *I. ricinus* and *I. persulcatus* ticks was observed, *D. reticulatus* microbiome composition appeared to be significantly different. Similarly to our observations, significant differences in microbial diversity and composition were reported in the study in the far-western United States between several *Ixodes*, *Dermacentor* and *Haemaphysalis leporispalustris* tick species [24]. 

There is strong evidence that ticks acquire a significant portion of their microbiome through exposure to their environment [25]; thus, the observed differences could be, at least partly, driven by specific tick-species-related ecological relationships and habitat parameters. Indeed, *I. ricinus* and *I. persulcatus* ticks occur in several ecoregions, commonly found in deciduous and coniferous woodland and mixed forests of European type, while *D. reticulatus* is a typical open country tick species, preferring meadows and pastures [8].

Recently, the important role of small-scale ecological variation and microbe–microbe interactions in shaping tick microbial communities was efficiently highlighted [7]. In our study, we looked at the possible microbiome correlation with different habitats, such as forest and woodland/grassy area ecotones. For *Ixodes* ticks, no such correlation was detected, while in *D. reticulatus,* a possible impact of the habitat type on the microbiome composition was observed. However, a small sample number of *D. reticulatus* ticks collected in the forest areas in this study should be acknowledged; thus, additional studies with larger sample sets should be conducted to verify this result.

Statistically significant differences in alpha and beta microbial diversity were observed between *Ixodes* tick life stages and sexes, with lower taxa richness indexes obtained for female ticks. This result is similar to other studies conducted in different tick species in Europe and America [24,26]. More specifically, a decrease in both microbial species richness and evenness was reported as the *Ixodes* tick matures from larva to adult [24,25]. Interestingly, this phenomenon was not apparently present in *D. reticulatus* ticks in our study, as no statistically significant differences in microbial composition were detected between life stages and sexes. These findings could be partly explained by the endosymbiont loads, as a high load of endosymbionts in *Ixodes* female ticks was detected. In contrast, *D. reticulatus* tick samples did not show the presence of a single dominant microbial species in either males, females or nymphs, indicating the unique physiology of this tick species or its interaction with the surrounding environment. The most abundant microbial genus in *D. reticulatus* ticks was *Francisella*, which was previously recognized as the endosymbiont for this tick species [27]. In addition, our results showed that the *Francisella* genus was closely associated with *D. reticula*tus ticks, while endosymbionts Ca. *Midichlorii* and Ca. *Lariskella* were associated with *I. ricinus* and *I. persulcatus* females, respectively. Indeed, Ca. *Midichloria mitochondrii* is a well-known main endosymbiont of the tick *I. ricinus,* which appears to be ubiquitous in females across the tick’s distribution, while a lower prevalence is observed in males [28]. In our study, the prevalence of Ca. *Midichloria* in *I. ricinus* females was 100% with an overall abundancy of 58.38%, while only a small fraction of sequencing reads in males and nymphs was attributed to this genus. Also, in females, the endosymbiont load negatively correlated with the presence of the *Rickettsia* genus, as a significantly higher abundance of Ca. *Midichloria* was observed in the *Rickettsia*-negative *I. ricinus* samples. This observation is in contrary to a recent study, where a positive correlation between Ca. *Midichloria* and the presence of *Rickettsia* was detected in *I. ricinus* nymphs [29]. However, the observed difference could be explained by the different tick stage; in addition, the *Rickettsia* genus was previously reported to be involved in antagonist relationships with pathogenic or symbiotic microbial genera in *I. ricinus* and *Rhipicephalus sanguineus* ticks [7,30]. On the other hand, a positive correlation between Ca. *Midichloria mitochondrii* load and *Rickettsia parkeri* presence in the tick *Amblyomma maculatum* was described recently [31]. Also, evidence of a competition between pathogenic and endosymbiotic *Rickettsia* species exists [32,33]. Unfortunately, the sequencing approach of our study, similarly to other high-throughput 16S-amplicon-sequencing-based studies, did not allow us to identify *Rickettsia* at the species level; thus, it is unclear whether the detected sequences belonged to pathogenic or parasitoid species. Additional studies are needed to address the question of the role and/or interplay of different *Rickettsia* species within tick microbiomes.

Another bacteria of the family *Candidatus Midichloriaceae*, Ca. *Lariskella arthropodarum* (provisionally named ‘*Montezuma*’), have been detected in *I. persulcatus* and *I. pavlovskyi* ticks, as well as in human blood in the Russian Far East [34,35,36]. The hypothesis about the loss of this endosymbiont during *I. persulcatus* male development has been proposed [37]; however, the role of Ca. *Lariskella* in the host ticks has not yet been clearly determined [38]. The previously reported Ca. *Lariskella* prevalence in adult *I. persulcatus* females in different studies in Russia and Estonia was very diverse, ranging from 8.4% up to 97.1% [35,37,39,40]. In our study, all *I. persulcatus* females were Ca. *Lariskella*-positive; however, a great variability in the endosymbiont load was observed. In contrary to *I. ricinus* females, there was no connection between the Ca. *Lariskella* load and the presence of *Rickettsia* genus. However, interestingly, significant differences in the microbiome composition, including Ca. *Lariskella* amounts, were observed between *I. persulcatus* female samples collected in different regions of Latvia. Previously, differences in the bacterial community structure and composition of ticks across habitats [8] and geographical sites [26] have been documented; thus, this result could indicate the existence of specific interactions between ticks, hosts and the surrounding environment within different ecological niches. 

## 5. Conclusions

The present study has assessed the microbiome composition of three endemic tick species in Latvia in the context of tick sex, geographical location, biotope and endosymbionts. Collectively, the obtained results revealed important associations between ticks and their microbial community and highlighted several tick microbiome features. We showed that, despite the sympatric occurrence of the tick species, tick microbiota and endosymbiont content were largely tick-species- and sex-specific, and were influenced by geographical location. Also, our data revealed possible interactions between *Rickettsia* and Ca. *Midichloria* in *I. ricinus* females. The obtained data provide the basis for additional studies to decipher the roles of, and interactions among, specific bacteria, which, in turn, could help to understand the factors that affect the tick microbial communities, and the consequences of microbiome variation. Further studies on the microbial communities of ticks within and between different ecosystems, and the influence of microbial diversity on the persistence and transmission of various medically important pathogens are of great importance.

## Figures and Tables

**Table 1:** Tick samples (N = 126).

Tick Species	Tick Stage	No (%) of Samples	Biotope, No. (%) of Tick Samples	
			Mixed Forest	Woodland/Grassy Area Ecotone
*I. ricinus*	Female	20 (37.7)	14 (70.0)	6 (30.0)
	Male	24 (45.3)	10 (41.7)	14 (58.3)
	Nymph	9 (17.0)	7 (77.8)	2 (22.2)
	Total	53	31 (58.5)	22 (41.5)
*I. persulcatus*	Female	16 (40.0)	10 (62.5)	6 (37.5)
	Male	17 (42.5)	7 (41.2)	10 (58.8)
	Nymph	7 (17.5)	4 (57.1)	3 (42.9)
	Total	40	21 (52.5)	19 (47.5)
*D. reticulatus*	Female	16 (48.5)	2 (12.5)	14 (87.5)
	Male	14 (42.4)	2 (14.3)	12 (85.7)
	Nymph	3 (9.1)	0	3 (100.0)
	Total	33	4 (12.1)	29 (87.9)
Total		126	56	70

## Data Availability

Raw sequencing reads have been submitted to the European Nucleotide Archive, project accession PRJEB63277.

## Supplementary Materials

The following supporting information can be downloaded at https://www.mdpi.com/article/10.3390/microorganisms11081970/s1, Figure S1: Tick collection sites in Latvia. Data were mapped using Google Earth. (A) I. *ricinus* samples, (B) I. *persulcatus* samples. Samples from Vidzeme region are encircled by blue, and samples from Latgale region are encircled by red line. (C) *D. reticulatus* samples. Figure S2: Quality assessment and sequence length control of 16S rRNA amplicons obtained in this study. Representative plots are shown. E7, E8, E10—tick samples; “-“—blank sample, bk—barcode used. The analysis was performed using a Bioanalyzer 2100 instrument with an Agilent High-Sensitivity DNA Kit (Agilent Technologies, United States). Figure S3: Taxonomically assigned sequencing reads per sample at the genus level. Figure S4: Clustering dendrogram based on the Bray–Curtis Index and the Ward clustering method. Samples with more similar genus profiles were clustered closer together. Table S1: Microbial genera detected in tick samples.
