# Supplementary material for: Comparative Microbiome Analysis of Three Epidemiologically Important Tick Species in Latvia
Source: Microorganisms. 2023 Jul 31;11(8):1970. doi: 10.3390/microorganisms11081970 (PMC10458549; doi:10.3390/microorganisms11081970)
Supplement: Supplementary file 1 [file microorganisms-11-01970-s001.zip › Suppl.Table_S1_abundance.pdf]

Supplementary Table S1. Microbial genera detected in tick samples.
The most abundant genera for each tick species/sex/developmental stage are indicated in bold.

	Female; I.ricinus	Male; I.ricinus	Nymph; I.ricinus	Female; I.persulcatus	Male; I.persulcatus	Nymph; I.persulcatus	Female; D.reticulatus	Male; D.reticulatus	Nymph; D.reticulatus
Acidibacter	0.08%	0.32%	1.71%	0.04%	0.12%	0.32%	0.09%	0.29%	0.00%
Acinetobacter	**0.96%**	2.05%	2.80%	0.19%	**5.15%**	2.06%	0.47%	1.10%	0.56%
Act_N09	0.01%	0.03%	0.25%	0.06%	0.69%	0.10%	0.01%	0.45%	0.00%
Actinomycetospora	0.04%	0.09%	0.04%	0.05%	0.05%	0.14%	0.40%	0.33%	0.69%
Aeromicrobium	0.04%	0.25%	0.04%	0.03%	0.08%	0.06%	0.14%	0.17%	0.27%
Agrobacterium	0.07%	0.30%	0.09%	0.16%	0.45%	0.29%	0.23%	0.24%	0.21%
Altererythrobacter	0.05%	0.11%	0.19%	0.05%	0.19%	0.10%	0.14%	0.20%	0.14%
Arthrobacter	0.52%	0.97%	0.71%	0.08%	0.47%	0.54%	0.23%	0.52%	0.21%
Aureimonas	0.13%	0.10%	0.04%	0.04%	0.01%	0.01%	0.28%	0.39%	0.75%
Borrelia	0.71%	0.27%	0.11%	**1.40%**	**7.88%**	0.41%	0.02%	0.05%	0.00%
Bosea	0.13%	0.68%	0.41%	0.31%	0.75%	0.10%	0.46%	0.89%	0.45%
Bradyrhizobium	0.26%	0.83%	**3.56%**	0.21%	0.47%	0.79%	0.41%	0.79%	0.34%
Brevundimonas	0.06%	0.20%	0.25%	0.14%	1.75%	0.17%	0.19%	0.26%	0.14%
Burkholderia	0.24%	0.40%	0.04%	0.11%	0.80%	0.11%	0.20%	0.63%	0.10%
Candidatus_Endoecteinascidia	0.00%	0.17%	0.02%	0.00%	0.00%	0.00%	**1.23%**	1.15%	0.69%
Candidatus_Lariskella	0.06%	0.01%	0.00%	**50.07%**	0.13%	**6.43%**	0.00%	0.00%	0.00%
Candidatus_Midichloria	**58.38%**	0.58%	2.04%	0.06%	0.14%	0.91%	0.02%	0.00%	0.00%
Candidatus_Neoehrlichia	0.12%	0.52%	0.00%	0.00%	0.58%	0.01%	0.00%	0.00%	0.00%
Caulobacter	0.10%	0.16%	0.44%	0.06%	0.56%	0.43%	0.11%	0.04%	0.10%
Caulobacteraceae_Group	0.18%	0.33%	1.63%	0.14%	0.55%	0.45%	0.28%	0.72%	0.06%
Clavibacter	0.10%	0.15%	0.09%	0.00%	0.01%	0.01%	0.04%	0.08%	0.05%
Cloacibacterium	0.16%	0.87%	0.42%	0.02%	0.35%	0.75%	0.56%	1.01%	0.06%
Comamonadaceae_Group	0.12%	0.46%	0.19%	0.11%	0.45%	0.20%	0.23%	0.27%	0.46%
Comamonas	0.12%	0.12%	0.59%	0.02%	0.49%	0.08%	0.06%	0.05%	0.06%
Corynebacterium	**0.92%**	2.60%	**3.16%**	0.62%	**5.15%**	**4.90%**	0.69%	**1.71%**	0.69%
Coxiellaceae_Group	0.01%	1.17%	0.01%	0.06%	0.10%	0.00%	0.05%	0.11%	0.00%
Cupriavidus	0.38%	0.68%	0.46%	0.04%	0.27%	0.29%	0.07%	0.08%	0.16%
Curtobacterium	0.60%	0.62%	0.18%	0.00%	0.03%	0.09%	0.04%	0.05%	0.18%
Devosia	0.56%	0.79%	0.27%	0.43%	0.75%	0.23%	0.36%	0.30%	0.27%
Ehrlichia	0.00%	0.01%	0.00%	0.00%	2.17%	0.00%	0.00%	0.00%	0.19%
Enhydrobacter	0.12%	0.83%	0.71%	0.06%	0.27%	0.73%	0.14%	0.71%	0.00%
Enterobacter	0.10%	0.19%	0.21%	0.18%	0.12%	0.12%	0.10%	0.11%	0.08%
Erythrobacter	0.03%	0.09%	0.09%	0.03%	0.11%	0.05%	0.08%	0.10%	0.06%
Francisella	0.08%	**5.14%**	0.49%	0.00%	0.00%	0.01%	**52.30%**	**39.57%**	**47.66%**
Frondihabitans	0.12%	0.11%	0.12%	0.00%	0.03%	0.01%	0.03%	0.05%	0.08%
Fulvimonas	0.18%	0.61%	0.05%	0.29%	0.49%	0.04%	0.07%	0.17%	0.03%
Haemophilus	0.07%	0.14%	0.41%	0.06%	0.19%	0.71%	**1.22%**	0.14%	0.06%
Halomonas	0.73%	**3.37%**	**7.80%**	**1.21%**	**5.00%**	**7.00%**	0.52%	**2.63%**	0.75%
Janthinobacterium	0.43%	0.50%	0.14%	0.07%	0.27%	0.08%	0.05%	0.10%	0.21%
Jatrophihabitans	0.06%	0.17%	0.07%	0.02%	0.07%	0.04%	0.37%	0.35%	**0.96%**
Kaistobacter	0.18%	0.36%	0.41%	0.11%	0.33%	0.14%	0.27%	0.38%	0.50%
Kocuria	0.13%	0.88%	1.83%	0.07%	0.46%	1.39%	0.18%	0.46%	0.08%
Legionella	0.06%	0.17%	0.02%	0.02%	0.08%	0.03%	0.53%	0.71%	0.21%
Leptothrix	0.05%	0.12%	0.30%	0.07%	0.29%	1.04%	0.02%	0.04%	0.48%
Limnobacter	0.12%	0.28%	0.55%	0.07%	0.74%	1.02%	0.16%	0.09%	0.61%
Limnohabitans	0.05%	0.24%	0.02%	0.07%	0.16%	0.02%	0.08%	0.12%	0.22%
Luteibacter	0.49%	1.44%	0.27%	**0.81%**	2.04%	0.12%	0.26%	0.42%	0.02%
Massilia	0.64%	0.43%	0.58%	0.07%	0.58%	0.29%	0.12%	0.21%	0.42%
Mesorhizobium	0.03%	0.18%	0.02%	0.15%	0.28%	0.07%	0.06%	0.16%	0.08%
Methylibium	0.06%	0.17%	0.14%	0.06%	0.15%	0.05%	0.20%	0.08%	0.45%
Methylobacterium	**1.20%**	**3.26%**	**4.21%**	0.25%	1.79%	**2.93%**	**2.51%**	**3.18%**	**5.12%**
Methylopila	0.03%	0.21%	0.04%	0.05%	0.18%	0.05%	0.30%	0.27%	0.06%
Microbacterium	0.23%	0.74%	1.27%	0.07%	1.08%	1.23%	0.29%	0.69%	0.53%
Micrococcus	0.55%	1.16%	2.29%	0.09%	1.10%	1.71%	0.19%	0.57%	0.30%
Mitochondria_Group	0.00%	0.79%	0.02%	0.04%	0.10%	0.05%	0.07%	0.02%	0.03%
Mycobacterium	**4.59%**	**9.85%**	2.12%	**2.40%**	**4.01%**	1.26%	**1.30%**	**1.58%**	**2.16%**
Mycoplana	0.02%	0.19%	0.11%	0.04%	0.18%	0.11%	0.09%	0.12%	0.13%
Nakamurella	0.16%	0.30%	0.14%	0.06%	0.05%	0.12%	0.40%	0.27%	0.37%
Nesterenkonia	0.06%	0.42%	1.77%	0.01%	0.03%	0.01%	0.63%	**1.84%**	0.03%
Nitrosomonadaceae_Group	0.04%	0.38%	1.51%	0.05%	0.13%	0.05%	0.11%	0.05%	0.11%
Nocardioides	0.34%	1.26%	0.59%	0.17%	0.51%	0.39%	**1.20%**	0.92%	**1.81%**
Novosphingobium	0.26%	0.78%	**4.92%**	0.18%	0.67%	0.13%	0.30%	0.68%	0.35%
Paenibacillus	0.05%	0.35%	0.49%	0.05%	1.22%	0.53%	0.14%	0.49%	0.18%
Paracoccus	0.23%	0.81%	1.03%	0.09%	0.67%	1.69%	0.80%	0.47%	0.50%
Pedobacter	0.05%	0.20%	0.07%	0.22%	0.45%	0.10%	0.10%	0.18%	0.35%
Pelomonas	0.21%	0.54%	1.64%	0.16%	1.45%	**4.31%**	0.16%	0.14%	**1.95%**
Phenylobacterium	0.14%	0.35%	0.24%	0.04%	0.63%	0.29%	0.26%	0.16%	0.34%
Phyllobacterium	0.09%	0.39%	0.01%	0.40%	0.60%	0.03%	0.03%	0.03%	0.00%
Propionibacterium	**1.25%**	**3.44%**	**5.30%**	**1.58%**	**5.71%**	**10.62%**	**1.20%**	**1.98%**	**1.01%**
Pseudoclavibacter	0.05%	0.07%	0.06%	0.04%	0.21%	0.12%	0.06%	0.24%	0.02%
Pseudomonas	**1.00%**	**3.14%**	1.27%	**5.18%**	**4.04%**	0.93%	**0.99%**	**1.90%**	0.40%
Pseudonocardia	0.18%	0.22%	0.30%	0.06%	0.14%	0.16%	0.24%	0.28%	0.10%
Ralstonia	0.06%	0.17%	0.41%	0.05%	0.38%	1.76%	0.01%	0.00%	0.35%
Rhizobium	0.18%	0.66%	0.26%	0.41%	0.98%	0.16%	0.16%	0.24%	0.14%
Rhodanobacter	0.08%	0.34%	0.17%	0.24%	0.36%	0.01%	0.09%	0.16%	0.00%
Rhodococcus	0.51%	1.18%	0.25%	0.11%	0.44%	0.30%	0.31%	0.38%	0.85%
Rhodocyclaceae_Group	0.03%	0.14%	0.04%	0.02%	0.06%	0.03%	0.85%	0.81%	0.10%
Rickettsia	**9.12%**	**7.82%**	**6.52%**	**18.19%**	**4.84%**	**8.90%**	**14.30%**	**10.29%**	**3.94%**
Rickettsiella	0.03%	**3.45%**	0.12%	0.20%	0.39%	0.00%	0.02%	0.07%	0.02%
Rothia	0.08%	0.25%	0.54%	0.06%	0.29%	0.97%	0.08%	0.30%	0.05%
Salinibacterium	0.07%	0.18%	0.04%	0.01%	0.06%	0.05%	0.08%	0.09%	0.06%
Sediminibacterium	0.06%	0.16%	0.44%	0.02%	0.44%	0.30%	0.07%	0.27%	0.24%
Singulisphaera	0.01%	0.07%	0.01%	0.01%	0.03%	0.03%	0.19%	0.72%	0.03%
Sinobacteraceae_Group	0.05%	0.15%	0.47%	0.03%	0.06%	0.24%	0.07%	0.12%	0.11%
Sphingomonadaceae_Group	0.22%	0.54%	0.47%	0.11%	0.53%	0.51%	0.33%	0.42%	**0.85%**
Sphingomonas	**4.88%**	**12.44%**	**11.67%**	**1.40%**	**6.65%**	**15.81%**	**3.64%**	**5.42%**	**11.67%**
Sphingopyxis	0.06%	0.29%	0.17%	0.06%	0.30%	0.13%	0.13%	0.20%	0.22%
Spiroplasma	0.10%	0.00%	0.00%	**7.41%**	**8.32%**	0.00%	0.29%	0.01%	0.00%
Staphylococcus	0.49%	1.73%	**3.36%**	0.47%	2.94%	**4.06%**	0.38%	1.14%	0.83%
Stenotrophomonas	0.11%	0.35%	0.04%	0.23%	0.18%	0.03%	0.29%	0.14%	0.05%
Streptococcus	0.48%	1.08%	**3.16%**	0.15%	0.73%	**2.48%**	0.86%	0.81%	0.16%
Streptophyta_Group	0.06%	0.20%	0.21%	0.08%	0.25%	0.27%	0.09%	0.24%	0.06%
Tepidimonas	0.14%	0.65%	1.78%	0.05%	0.42%	1.67%	0.38%	0.43%	0.42%
Unclassified	1.15%	4.47%	5.19%	1.31%	3.38%	2.19%	1.60%	2.61%	2.98%
Variovorax	0.14%	0.74%	0.14%	0.20%	0.67%	0.12%	0.37%	0.34%	0.61%
Williamsia	**2.31%**	**2.86%**	1.13%	0.08%	0.12%	0.19%	0.37%	0.27%	0.64%
