# Supplementary figures and images for: Comparative Microbiome Analysis of Three Epidemiologically Important Tick Species in Latvia
Source: Microorganisms. 2023 Jul 31;11(8):1970. doi: 10.3390/microorganisms11081970 (PMC10458549; doi:10.3390/microorganisms11081970)

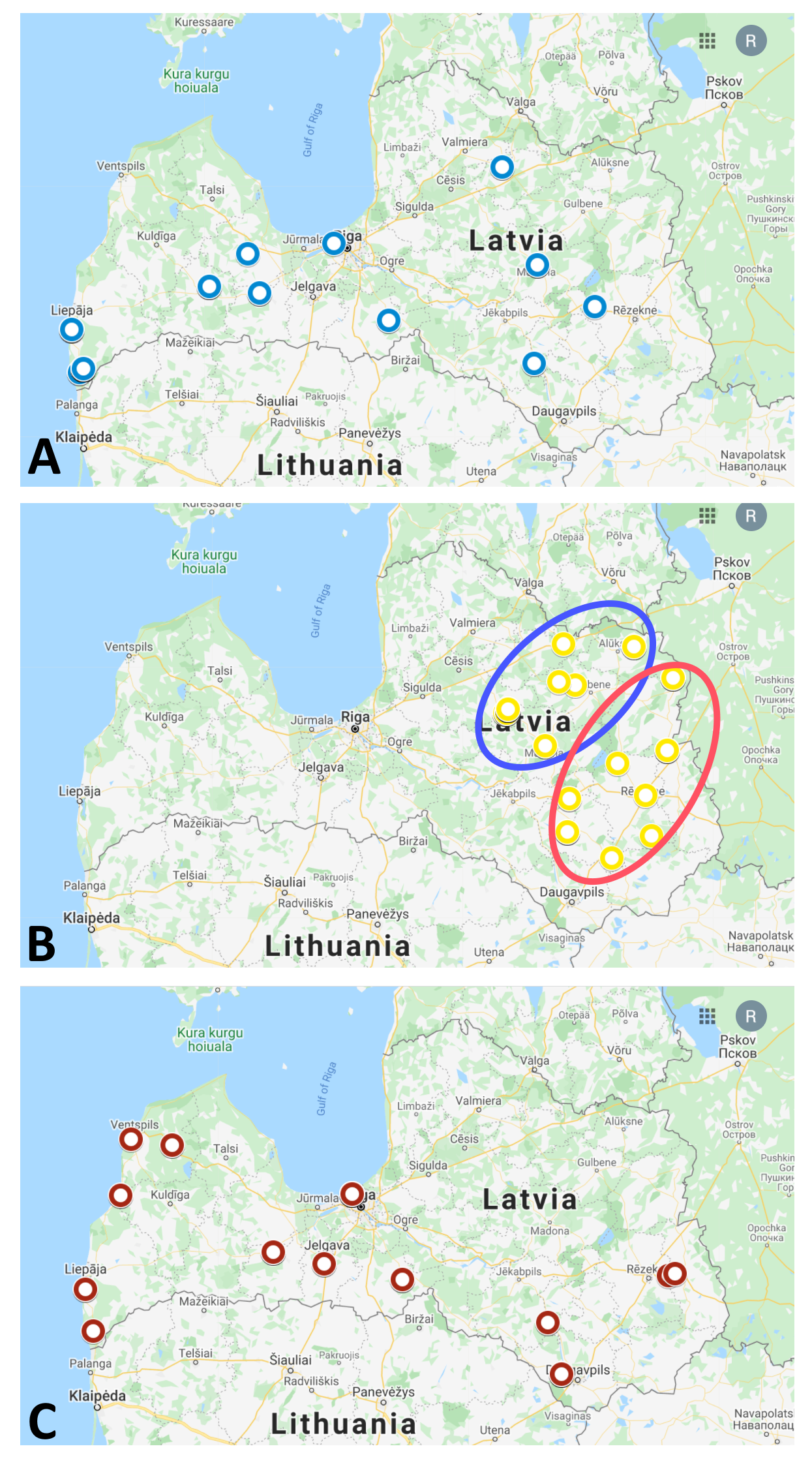

Supplement: Supplementary file 1 [file microorganisms-11-01970-s001.zip › Suppl_Figure_S1_Tick_Map2.tif]

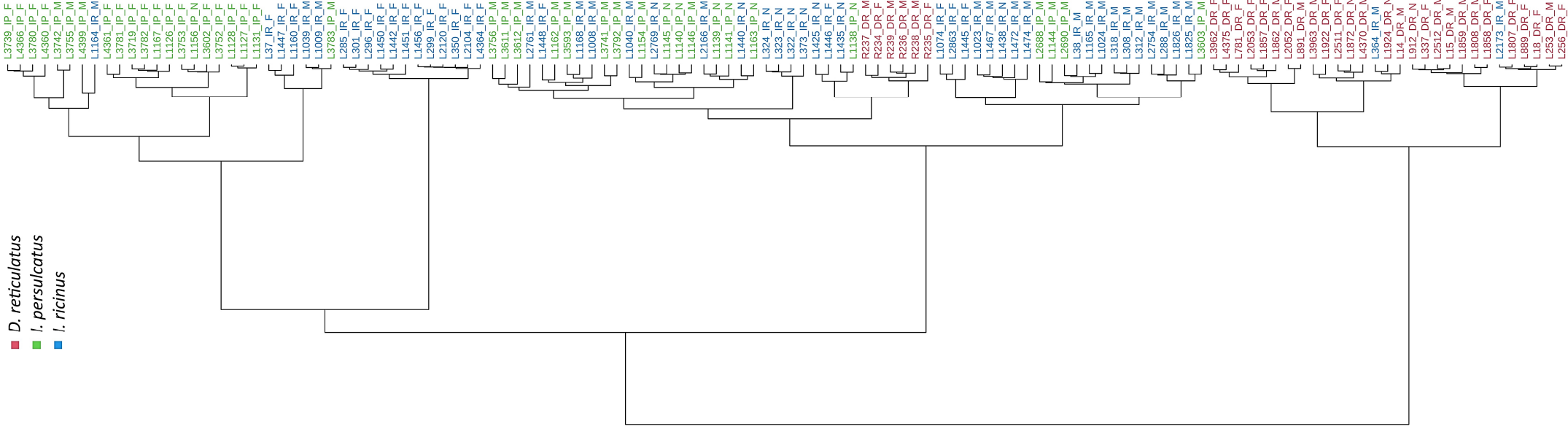

Supplement: Supplementary file 1 [file microorganisms-11-01970-s001.zip › Suppl_Figure_S4_.pdf]

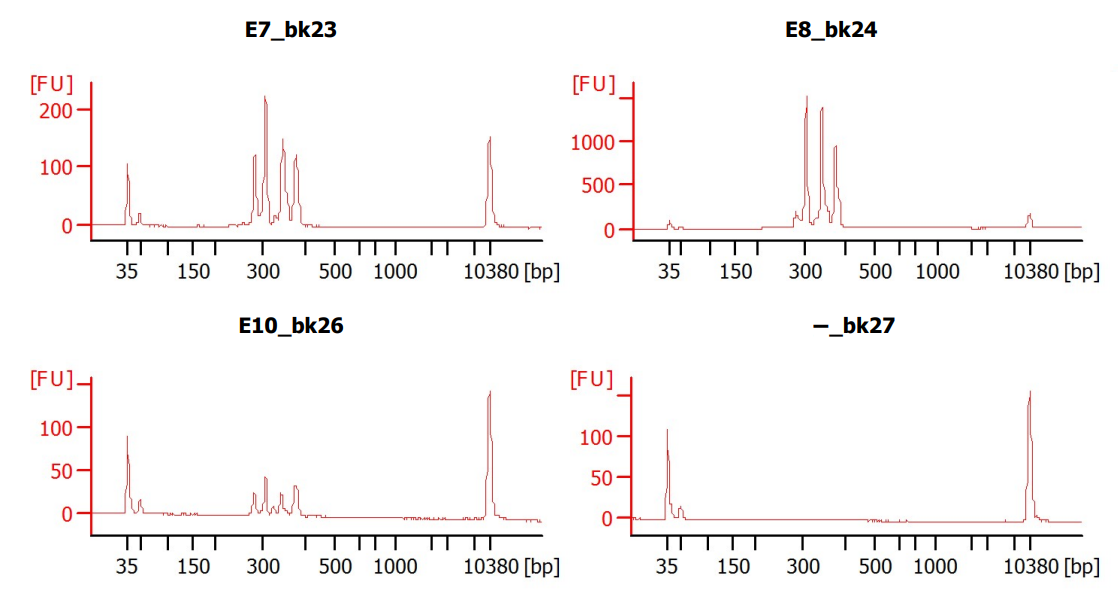

Supplement: Supplementary file 1 [file microorganisms-11-01970-s001.zip › Suppl_Fig_S2_blank.tif]

Library Size Overview

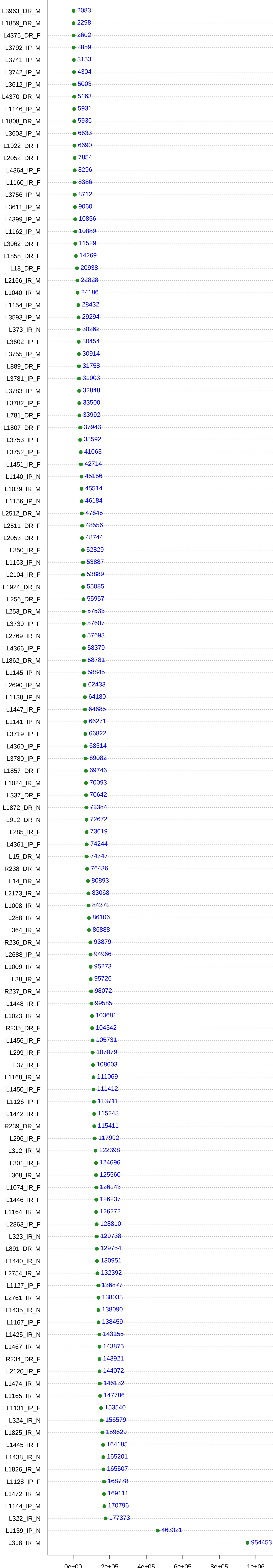

Supplement: Supplementary file 1 [file microorganisms-11-01970-s001.zip › Suppl_Fig_S3_norm_libsizes_.pdf]
